# Response of masticatory muscles to treatment with orthodontic aligners: a preliminary prospective longitudinal study

**DOI:** 10.1590/2177-6709.28.1.e232198.oar

**Published:** 2023-04-14

**Authors:** Sylvia de Araújo PAES-SOUZA, Marco Antonio Cavalcanti GARCIA, Victor Hugo SOUZA, Liliane Siqueira MORAIS, Lincoln Issamu NOJIMA, Matilde da Cunha Gonçalves NOJIMA

**Affiliations:** 1 Universidade Federal do Rio de Janeiro, Departamento de Odontopediatria e Ortodontia, Programa de pós-graduação em Odontologia (UFRJ, Rio de Janeiro/RJ, Brazil).; 2 Universidade Federal de Juiz de Fora, Departamento de Fisiologia do ICB, Programa de pós-graduação em Ciências da Reabilitação e Desempenho Físico Funcional (UFJF, Juiz de Fora/MG, Brazil).; 3 Aalto University, Department of Neuroscience and Biomedical Engineering (Espoo, Finland).

**Keywords:** Masticatory muscles, Electromyography, Clear aligners

## Abstract

**Introduction:**

The emergence of orthodontic aligners has provided an aesthetic and comfortable option for orthodontic treatment. However, the encapsulated design of the aligners can influence the masticatory muscles, and might compromise safe treatment.

**Objective:**

This preliminary longitudinal study aimed to investigate whether the use of orthodontic aligners affects the biting force and myoelectric activity of the superficial masseter and anterior temporal muscles.

**Methods:**

Ten subjects participated in the study and underwent treatment during an 8-month follow-up period. The root mean square (RMS), the median power frequency (MPF) of the surface electromyography (sEMG) signals, and the biting force (kgf) were recorded and normalized relative to the pretreatment condition. The data were analyzed by repeated-measure analysis of variance (ANOVA), with the significance level set at 5%.

**Results:**

Both the superficial masseter and the anterior temporal muscles presented an increase in sEMG signal activity during the treatment, with a marked increase in the latter compared to the former (*p*<0.05). Moreover, a significant decrease in bite force was evidenced (*p*<0.05).

**Conclusions:**

This preliminary study observed that the orthodontic aligners affected the muscle recruitment pattern of masticatory muscles, and reduced biting performance during the 8-month follow-up period.

## RESUMO

**Introdução:**

O surgimento dos alinhadores ortodônticos tem proporcionado uma opção estética e confortável para o tratamento ortodôntico. No entanto, o *design* encapsulado dos alinhadores pode influenciar os músculos mastigatórios e comprometer a segurança do tratamento.

**Objetivo:**

Este estudo longitudinal preliminar teve como objetivo investigar se o uso de alinhadores ortodônticos afeta a força de mordida e a atividade mioelétrica dos músculos masseter superficial e temporal anterior.

**Métodos:**

Dez indivíduos participaram do estudo e foram submetidos a tratamento durante um período de acompanhamento de 8 meses. A raiz quadrada média (RMS), a frequência mediana de potência (FMP) dos sinais de superfície da eletromiografia (sEMG) e a força de mordida (kgf) foram registradas e normalizadas em relação à condição de pré-tratamento. Os dados foram analisados por análise de variância para medidas repetidas (ANOVA), com nível de significância estabelecido em 5%.

**Resultados:**

Tanto o masseter superficial quanto o temporal anterior apresentaram aumento da atividade do sinal sEMG durante o tratamento, com aumento acentuado desse último em comparação ao primeiro (*p*<0,05). Além disso, foi evidenciada uma diminuição significativa da força de mordida (*p*<0,05).

**Conclusões:**

Esse estudo preliminar observou que os alinhadores ortodônticos afetaram o padrão de recrutamento muscular dos músculos mastigatórios e reduziram o desempenho da mordida durante o período de acompanhamento de oito meses.

## INTRODUCTION

The relevance of aesthetic values is reflected by the popularity of orthodontic aligners as a viable therapeutic approach to meet society’s current demands.^1^ However, the short and long-term effects of this new therapy on mastication biomechanics are still unclear.

The encapsulated occlusal devices used for bruxism and temporomandibular disorders (TMD) have a myorelaxant effect on masticatory muscle activity.^2^ Unlike the occlusal devices also used in the treatment of bruxism, encapsulated orthodontic retainers used in the short-term have not been found to cause changes in muscle recruitment.^3^ However, orthodontic retainers and orthodontic aligners cannot be compared with occlusal devices, despite their similar encapsulated design, because aligners are usually not adjusted for more stable occlusal contact, during clinical practice.

The Invisalign^®^ system (Align Technology, San Jose, CA, USA) is a device with a 0.7-mm thickness covering the clinical crown of the dental elements and adjacent gingiva.^4^ This encapsulated design can modify the standard posture during the dental rest condition and salivary swallowing, leading to changes in the masticatory muscle recruitment pattern.

Quantitative analysis of the bite force produced is also a well-known clinical parameter used to assess chewing performance. Neuromuscular adaptation in bite force has been reported in the orthodontic retention phase.^6^ Treatment with Invisalign^®^ has been found to help reduce the painful symptomatology of TMD patients.^7^ Moreover, the use of aligners in individuals with sleep bruxism (SB) did not influence the SB index, and was able to increase masseter phasic contractions.^8^ An increase in the myoelectric activity of the masseter after short periods of aligner use has also been reported.^9^ However, to our knowledge, there is no previous account of the effect of orthodontic aligners used for long-term periods on the response of other masticatory muscles during different tasks in asymptomatic subjects.

To this end, this preliminary longitudinal study aimed to investigate whether orthodontic aligners can affect the biting force and myoelectric activity of superficial masseter and anterior temporal muscles. The null hypothesis tested was that separation of the dental arches and lack of occlusal adjustment prompted by the aligner would change mastication muscle recruitment during orthodontic treatment with Invisalign. 

## MATERIAL AND METHODS

This prospective longitudinal clinical study included 10 participants (7 women; mean age 29.9 ± 5.5 years). This experimental protocol for the study was approved by the local ethical committee (process number 2.096.512/2017), following the principles of the Declaration of Helsinki. All the subjects gave their written informed consent to participate in the study. The selected subjects had Angle Class I or Class II malocclusions, crowding of teeth ≤ 5.0 mm, a good vertical facial pattern (SN.GoGn = 27º to 37º), and a functional state of general and periodontal health. The individuals who were excluded comprised those under orthodontic treatment, and indicated for tooth extraction or auxiliary mechanics (mini-implants, mini-plates, buttons, precision cuts or intermaxillary elastic), or else affected by syndromes with blood-related or dentofacial manifestations, temporomandibular joint dysfunctions, crossbite or open bite, and reporting routine use of analgesics, anti-inflammatory drugs, muscle relaxants or anxiolytics. 

All participants were instructed to use the aligners for at least 22 hours daily. The following pairs of aligners were changed every two weeks after that, as defined in the treatment plan. The time of daily use and the duration of each pair of aligners for 14 days followed the manufacturer’s instructions.

Surface electromyography (sEMG) was used to evaluate the bilateral recruitment of superficial masseter and anterior temporal muscles. The sEMG signals were recorded at predefined time intervals during an 8-month follow-up period. The initial exam (T0) was performed one week before using the first pair of aligners, and defined as the baseline condition (pretreatment muscle parameters). The sEMG signal was recorded with and without the aligners in the buccal cavity, during the procedure and after the beginning of the treatment (T1-T8). The experimental assessment was performed on the day in which the first pair of aligners was installed (T1), and the subsequent assessments, 1, 2, 4, 8, 16, 24 and 32 weeks after T1, labeled as T2, T3, T4, T5, T6, T7, and T8, respectively.

The sEMG signals were recorded using surface electrodes (Ag-AgCl) (Meditrace Kendall, REF 31118733 - Covidien™ Brazil), and digitized with a 5-channel EMG System^®^ (model EMG800C-532, São José dos Campos/SP, Brazil; 16-bit A/D conversion; 2 kHz sampling rate per channel; a band-pass filter [Butterworth 4th order]: 20-500 Hz; gain: 2000; and common-mode rejection rate: ≥ 100 dB). Four channels were used to acquire the sEMG signals, and one channel was used to measure the bite force with a mandibular dynamometer (maximum capacity: 200 kgf) by EMG System^®^. The sEMG signal acquisitions were performed using the EMG Lab software program (version 1.1/2012, EMG System®, São José dos Campos/SP, Brazil).

**Figure 1:**
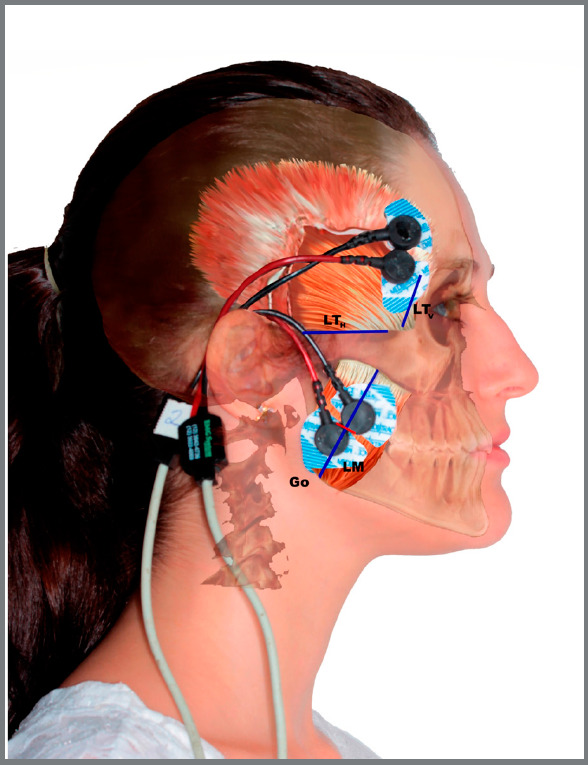
Schematic illustration based on clinical photography and cone-beam computed tomography of a participant, depicting the craniofacial complex in the lateral view. The reference lines (LT_V_: Vertical line of anterior temporal muscle; LT_H_: Horizontal line of anterior temporal muscle, and LM: masseter line) are used to position the surface electrodes on the superficial masseter and anterior temporal muscles. The red line represents the point located at 40% length of the LM, from the gonion point. Note the direction of the muscle fibers and the area of the superficial masseter muscle tendon.

The electrodes were placed according to the protocol suggested by Sabaneeff et al,^10^ as shown in Figure 1. The positioning was chosen according to the methodology used, and was recorded on a clinical form, in the first step of the data collection procedure, to confirm the correct positioning for the exams.

The sEMG signals were recorded at three recruitment levels: mandibular rest position, maximum voluntary bite force (MVBF), and submaximal voluntary bite force (SVBF). Three acquisitions were performed in MVBF, and the median value was obtained from the mandibular dynamometer. The 30% MVBF was calculated and defined for the third task, i.e., SVBF. The raw sEMG signals were recorded during the three recruitments levels, whenever measurements were made (T0-T8), and each recording lasted 20 seconds.

The sEMG signals were processed by the SignalHunter software program^11^ written in MATLAB R2015a (Math- Works, Natick, MA, USA). A 2-s window was extracted from the 10th second of the sEMG signal acquisition, to record data in the mandibular rest position, and another 2-s window was selected from the dynamometer to assess the MVBF and SVBF conditions. The chosen window was visually placed where the MVBF or the SVBF shown by the dynamometer reached an approximately constant level, and had the lowest variability. All the sEMG procedures adopted in this study followed the SENIAM recommendations.^12^

Signal analysis was performed in the time and frequency domains. The sEMG root mean square (RMS) amplitude was estimated with the following equation:

$$RMS=\sqrt{\frac{1}{N}\sum_{n=1}^{N}EMG{\left[n\right]}^{2}}$$

N represents the number of samples (= 4000) at the analyzed intervals. 

The median frequency was also extracted from the power spectrum of the sEMG signal using the following equation:

$$\sum_{f=0}^{MDF}P(f)=0.5$$

In which f indicates the frequencies represented in the energy power spectrum (P) of the sEMG signal, separating two regions of similar power.

The RMS amplitude and the median power frequency (MPF) were calculated for all the muscles evaluated, and from the 2-s window selected from the signal referring to the dynamometer channel.

## STATISTICAL ANALYSIS

The Shapiro-Wilk test was used to assess whether the data distribution was Gaussian. Based on the results of the normality test, the data were analyzed using parametric tests. RMS amplitude, MPF, and bite force (kgf) were normalized relative to the baseline value (T0) for all three recruitment levels. The effects related to the recruitment level (mandibular rest, MVBF and SVBF), the side (right vs. left), and the use or non-use of an aligner (with aligner [WA] vs. without aligner [WoA]) on the RMS amplitude, MPF and force were performed using repeated-measures analysis of variance (ANOVA). 

The normality of residuals and data homoscedasticity were verified to meet the ANOVA assumptions. The comparison of RMS amplitude and MPF was performed separately for each muscle. The Bonferroni *post-hoc* test was used for multiple comparisons whenever necessary. The level of significance was set at 0.05.

## RESULTS

**Figure 2:**
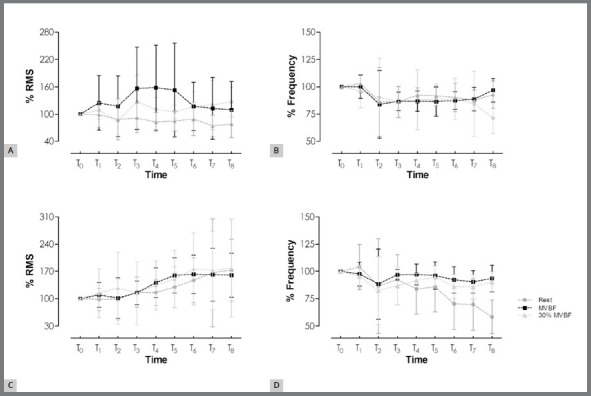
(**A**) Normalized RMS amplitude and (**B**) mean median frequency on both sides of the superficial masseter muscle for the whole treatment period; (**C**) normalized RMS amplitude and (**D**) mean median frequency of both sides of the anterior temporal muscle during the entire treatment period. Error bars represent the standard deviation.

The data of the descriptive statistics of the muscles studied, the side and the level of recruitment during the eight months of follow-up are available in Table 1. The RMS amplitude variation for the superficial masseter muscle is shown in Figure 2A. The sEMG activity increased at the end of the evaluation period (~30% for the RMS value; F_(8, 736)_=2.72; *p*=0.001), and throughout the treatment in conditions of rest. RMS amplitude for MVBF increased during the initial evaluation (T1-T3), but was followed by a gradual decrease over time, resulting in a ~20% increase toward the end (T8). The RMS amplitude in SVBF decreased about 20% from pretreatment (T0) to the end of follow-up (T8). The data obtained from the MPF values (T0 to T3-T8) (F_(8, 736)_=2.72; *p*=0.001) for all tasks decreased by about 30%, following the opposite variation observed in the RMS amplitude (Fig 2B). 

**Table 1:** Median and standard deviation of both the normalized data of the root mean square (RMS amplitude), and the median power frequency (MPF) of the superficial masseter muscles and anterior temporalis. The data referred to the three tasks of muscular activity, their respective hemifaces, and their variations over the 8-month follow-up period.

TASKS	T1	T2	T3	T4	T5	T6	T7	T8
RIGHT MASSETER - RMS (%)								
REST WITHOUT ALIGNER	116.3±39.9	80.7±31.8	153.8±71.6	122.9±44.6	106.7±33.8	156.4±98.2	182.3±129.4	198.0±99.1
REST WITH ALIGNER	139.5±79.5	96.5±54.6	116.3±33.0	129.1±68.8	113.8±29.4	158.3±99.0	152.8±185.4	132.8±56.0
MVBF WITHOUT ALIGNER	113.3±42.5	119.5±78.9	135.2±61.7	177.3±146.4	141.6±97.6	106.5±45.0	87.5±48.6	112.8±76.5
MVBF WITH ALIGNER	148.4±100.9	114.6±68.1	162.7±105.6	154.5±71.8	164.2±131.9	110.0±49.6	103.6±52.6	114.0±74.3
SVBF WITHOUT ALIGNER	113.8±46.8	95.3±50.7	112.6±44.7	94.5±36.5	88.8±32.6	94.1±44.8	65.5±21.9	92.8±42.5
SVBF WITH ALIGNER	108.6±40.8	86.7±47.0	92.1±49.0	92.2±36.7	84.3±33.3	92.7±47.3	67.3±23.0	88.7±51.3
LEFT MASSETER - RMS (%)								
REST WITHOUT ALIGNER	100.6±14.0	86.5±32.1	110.8±57.2	100.1±19.3	102.1±22.8	102.9±22.3	244.0±405.2	109.6±24.9
REST WITH ALIGNER	95.2±21.9	86.1±34.3	134.0±71.4	102.6±25.2	98.2±18.6	106.0±21.7	259.1±497.6	126.6±45.5
MVBF WITHOUT ALIGNER	101.8±31.5	116.4±67.0	147.6±84.8	144.0±94.9	137.0±78.0	124.6±52.6	116.0±79.0	101.3±56.1
MVBF WITH ALIGNER	138.1±126.5	101.8±60.4	136.2±92.9	115.31±79.02	136.9±105.2	110.7±63.4	110.4±70.1	95.2±52.8
SVBF WITHOUT ALIGNER	88.7±26.4	85.1±42.3	92.3±42.3	71.1±20.6	83.8±21.4	80.6±29.2	74.9±31.8	69.5±21.2
SVBF WITH ALIGNER	87.8±20.8	83.8±43.3	77.9±32.8	77.6±21.2	88.5±34.4	80.7±39.3	81.2±32.1	68.1±23.0
RIGHT MASSETER - MEDIAN POWER FREQUENCY (%)								
REST WITHOUT ALIGNER	88.1±26.3	96.6±40.5	84.0±26.5	78.1±29.5	85.63±20.6	77.0±32.8	78.8±32.7	57.6±19.5
REST WITH ALIGNER	90.8±21.2	95.1±41.4	87.4±19.6	94.8±42.1	89.5±33.4	88.1±45.3	83.2±31.2	76.2±20.8
MVBF WITHOUT ALIGNER	98.3±12.9	82.0±35.1	84.2±14.4	85.1±18.7	83.9±18.0	86.7±11.6	91.0±15.8	98.1±19.0
MVBF WITH ALIGNER	97.9±12.3	86.6±31.7	84.5±15.3	84.7±14.5	83.0±17.9	86.0±10.3	89.2±14.5	96.2±18.2
SVBF WITHOUT ALIGNER	107.2±11.6	87.7±32.9	92.1±10.5	92.6±91.8	92.0±9.4	92.5±14.5	92.0±12.9	94.3±19.9
SVBF WITH ALIGNER	101.3±9.2	88.4±32.8	81.0±29.6	91.9±8.9	90.9±11.9	88.6±14.4	92.9±13.7	93.5±17.2
LEFT MASSETER - MEDIAN POWER FREQUENCY (%)								
REST WITHOUT ALIGNER	101.6±16.9	86.0±32.1	91.7±19.4	84.4±19.4	90.2±17.8	98.1±20.0	96.3±43.9	74.1±20.2
REST WITH ALIGNER	103.8±26.6	83.0±36.5	82.6±23.5	94.7±15.5	89.3±17.0	92.2±27.4	79.1±21.0	76.9±16.5
MVBF WITHOUT ALIGNER	104.2±21.1	83.1±30.0	88.0±9.2	86.4±7.7	90.2±12.8	88.7±9.0	88.8±14.1	99.1±12.8
MVBF WITH ALIGNER	100.5±13.3	87.5±31.9	89.1±7.4	91.8±14.3	88.0±10.6	88.8±10.8	86.5±5.95	95.2±8.2
SVBF WITHOUT ALIGNER	103.5±12.1	84.9±31.1	90.2±6.9	92.4±7.8	92.4±12.0	89.5±15.2	83.4±15.3	93.2±11.1
SVBF WITH ALIGNER	99.6±10.9	83.7±30.1	81.0±29.6	92.5±9.9	91.6±13.5	90.0±13.8	82.3±15.0	90.4±10.1
RIGHT TEMPORAL - RMS (%)								
REST WITHOUT ALIGNER	108.2±30.8	102.1±49.9	131.8±52.9	135.6±48.7	155.2±74.7	206.4±84.2	238.3±330.3	155.0±61.6
REST WITH ALIGNER	103.8±48.2	95.8±54.4	102.0±37.5	105.7±49.7	99.3±41.0	136.9±61.3	235.9±389.9	138.3±74.4
MVBF WITHOUT ALIGNER	113.3±38.3	112.0±66.4	116.0±37.5	149.2±47.7	160.3±48.0	154.8±29.6	149.9±56.8	165.4±57.9
MVBF WITH ALIGNER	113.7±35.9	109.8±65.3	122.4±40.0	147.2±43.6	172.0±52.8	156.6±35.2	162.4±70.2	159.7±56.4
SVBF WITHOUT ALIGNER	139.5±112.9	141.7±121.2	141.4±133.2	148.2±86.0	182.0±128.6	205.0±147.2	202.4±179.5	215.4±208.4
SVBF WITH ALIGNER	140.4±118.9	144.8±154.7	132.7±143.7	171.4±126.1	163.0±120.0	199.4±123.8	179.0±109.3	204.7±178.9
LEFT TEMPORAL - RMS (%)								
REST WITHOUT ALIGNER	97.6±38.5	113.6±63.3	139.9±63.1	134.0±66.9	175.5±116.8	132.6±53.7	175.7±129.5	300.0±307.4
REST WITH ALIGNER	82.9±18.7	83.6±53.3	92.9±34.0	88.3±35.4	88.2±31.2	199.4±326.5	157.2±160.6	184.5±89.8
MVBF WITHOUT ALIGNER	107.4±40.5	92.8±49.1	108.8±32.3	132.0±45.0	147.9±51.7	168.3±67.4	164.8±75.9	154.7±62.1
MVBF WITH ALIGNER	109.2±36.6	93.5±45.0	111.2±26.8	141.7±37.0	165.1±55.4	163.4±78.7	173.4±84.2	157.4±68.3
SVBF WITHOUT ALIGNER	92.1±26.9	131.6±126.0	98.1±29.7	104.4±39.2	124.4±40.5	154.1±50.2	142.7±75.4	152.2±68.7
SVBF WITH ALIGNER	86.8±17.1	91.2±41.0	84.8±42.6	112.2±30.0	131.5±47.02	147.3±72.5	164.4±78.3	143.2±68.0
RIGHT TEMPORAL - MEDIAN POWER FREQUENCY (%)								
REST WITHOUT ALIGNER	92.3±24.7	80.6±43.4	78.6±22.0	69.3±30.2	65.8±19.1	54.2±19.7	73.7±33.5	64.4±24.1
REST WITH ALIGNER	94.6±31.6	78.8±44.0	88.8±40.1	68.6±23.4	93.3±39.4	67.4±37.1	60.0±28.9	67.3±23.3
MVBF WITHOUT ALIGNER	97.6±9.4	89.0±33.4	99.9±9.5	96.9±11.7	96.9±15.9	93.5±11.7	94.7±13.7	95.1±18.3
MVBF WITH ALIGNER	98.8±15.8	86.4±32.9	92.8±9.9	97.5±15.4	94.5±17.5	91.0±15.5	90.6±13.2	94.2±13.8
SVBF WITHOUT ALIGNER	97.0±14.0	84.7±32.8	94.5±15.2	92.0±12.7	95.7±17.8	88.4±15.6	88.6±15.4	93.0±18.6
SVBF WITH ALIGNER	95.5±13.1	82.9±31.2	83.7±32.0	90.5±13.4	98.5±28.5	86.7±19.7	86.1±18.2	89.1±16.9
LEFT TEMPORAL - MEDIAN POWER FREQUENCY (%)								
REST WITHOUT ALIGNER	111.3±32.4	81.2±32.4	89.1±20.3	87.9±33.4	76.7±22.8	83.2±35.9	77.1±30.6	49.4±23.1
REST WITH ALIGNER	117.8±31.5	105.7±56.0	113.2±35.0	109.6±47.1	107.2±43.6	76.4±39.0	68.0±25.8	51.9±22.8
MVBF WITHOUT ALIGNER	97.7±10.0	90.5±33.9	98.3±4.62	99.4±8.5	98.2±14.0	93.3±12.5	88.9±10.7	92.8±10.0
MVBF WITH ALIGNER	96.1±15.3	87.7±32.5	96.6±8.3	95.1±9.4	95.3±12.2	91.0±14.3	87.4±11.4	91.8±12.0
SVBF WITHOUT ALIGNER	95.7±9.4	77.6±33.3	91.2±6.2	93.1±10.2	92.7±9.6	89.9±11.9	88.2±17.3	92.1±10.5
SVBF WITH ALIGNER	9.3±7.1	85.9±30.9	78.8±28.1	92.7±6.9	94.6±12.6	84.8±20.9	84.4±13.1	90.2±13.3
BITE FORCE - kgf (%)								
WITHOUT ALIGNER	106.1±33.8	87.7±46.5	89.3±22.6	95.49±24.3	99.6±32.5	89.4±27.6	83.9±27.9	79.8±23.1
WITH ALIGNER	109.7±48.0	95.6±46.8	89.7±38.5	91.4±40.9	95.0±40.9	81.3±42.8	84.1±38.4	76.8±35.7
SUBMAXIMAL BITE FORCE - kgf (%)								
WITHOUT ALIGNER	104.1±14.9	84.4±35.4	86.0±11.1	86.9±16.2	91.6±19.7	88.8±25.7	73.0±17.9	89.4±41.5
WITH ALIGNER	94.7±19.8	88.9±23.6	75.6±36.8	83.1±23.8	86.7±27.7	77.9±34.8	75.9±26.8	71.5±26.80

There was a significant increase in the RMS amplitude (F_(8, 728)_=24.675; *p*=0.000) for the anterior temporal muscle, gradually from T0 to T4-T8 for all tasks (Fig 2C). At the end of the follow-up period, muscle recruitment increased by about 80% for the mandibular rest task, 70% for the MVBF, and 90% for the SVBF task. The MPF (F_(8, 752)_=17.119; *p*=0.000) also decreased by about 30%, following the opposite variation observed in the RMS amplitude (Fig 2D).

**Figure 3:**
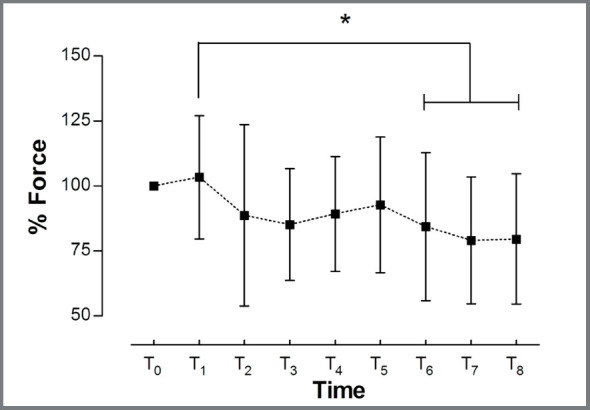
Normalized force of MVBF and SVBF during the whole treatment period (from T0 to T8); *p<0.015. Error bars represent the standard deviation.

The mean level of bite force decreased significantly (~20%), from T0 to T6-T8 (F_(8, 264)_=7.42; *p*<0.05) (Fig 3). No interaction was observed between the recordings performed with or without aligners (T1-T8) in the buccal cavity in the RMS amplitude (F_(8, 736)_=0.84; *p*=0.562) and MPF (F_(8, 752)_=0.94; *p*=0.481) for the superficial masseter, as well as the RMS amplitude (F_(8, 728)_=0.42; *p*=0.905) and MPF (F_(8, 752)_=1.79; *p*=0.074) for the anterior temporal muscles.

## DISCUSSION

This longitudinal prospective study aimed to evaluate the orthodontic biomechanical effects of aligners on the masticatory muscles during orthodontic treatment. The present preliminary results suggested changes in muscle recruitment strategies, corroborated by alterations in both temporal and spectral parameters of the sEMG signal, and by a decrease in bite force. 

It is well known that the neuromuscular activation pattern depends on the specific task that the muscle undertakes to develop. The masseter muscle function promotes mandibular closure, and provides greater isometric force during maximum clenching.^5^ This muscle is minimally recruited during mandibular rest, and is markedly called into play as the mandible closes.^13^ The present results showed that the myoelectric activity of the superficial masseter muscle increased relative to the baseline (T0), followed by a decrease in the median frequency content in the sEMG signal. Lou et al^9^ also observed an increase in the sEMG activity of the masseter muscle following the use of aligners, and a consequent decrease after four weeks of use. Our follow-up period was longer; it recorded the highest averages of myoelectric activity after 4 weeks of use, and a decrease in activity after 16 weeks of use. In this case, the present results suggest a hypothetical reorganization of the synergic pattern of masseter muscles in controlling the mandible position.

In turn, the function of the anterior temporal muscle is marked by mandibular balance and posture maintenance, and is more sensitive to changes in dental occlusion. During rest, the anterior temporal muscle also presents minimal recruitment. Despite the minimum recruitment of both muscles during rest, the anterior temporal muscle contributes more significantly for maintaining postural balance than the masseter muscle.^13^ In the present study, the anterior temporal muscle also showed an increase in sEMG signal amplitude, compared to the baseline values, even during rest. It is interesting to highlight that the sEMG signal (RMS) amplitude increased by about 70% from the baseline (T0) to the end of the treatment (T8), in comparison with all muscle recruitment levels studied herein (mandibular rest position, MVBF, and SVBF). We may conjecture that this increase resulted from the lack of occlusal stability, which overloaded the postural balance of the jaw muscle.

Significant changes in the myoelectric activity of orthodontic aligner usage were identified over time, concerning the baseline values of each subject. Non-synergistic masticatory muscle activation has been observed in previous studies.^14^ ^-^ ^16^ According to the present results, the use of the orthodontic aligners presumably led to new activation patterns adopted to control the stomatognathic system. The difference in the muscle recruitment pattern suggests that the biomechanics of the stomatognathic system was upset when greater recruitment was required of the temporal muscle in tasks that are usually performed in conjunction with superficial masseter. Based on this assumption, we can presume that the increase in the myoelectric activity of the anterior temporal muscle may be attributed to a reorganization in the protective reflex of the masseter muscle to prevent the teeth from being damaged by excessive bite force; however, the authors recognize that this hypothesis needs to be further examined. The increase in relative RMS amplitude exhibited by both muscles during mandibular rest and contraction suggests that additional recruitment was required to keep the system in balance.

Periodontal pressoreceptors are known to provide feedback to the chewing muscles.^17^ It can be expected that periodontal mechanoreceptors will be continuously activated during bite tasks, thus allowing larger individual motor units to be recruited quickly, thereby developing larger closing forces without much effort.^18^ We can speculate that the encapsulated design of the aligners and the application of orthodontic force generated by the different attachments may have recruited individual afferent periodontal mechanoreceptors in distinct directions, and influenced the motor output of the trigeminal nerve in the closing muscles, thus leading to increased muscle recruitment. 

The polyurethane composing the structure of the Invisalign^®^ orthodontic aligners is amorphous and/or semi-crystalline, with types of spatial connections and arrangements that function as either a rigid or a resilient substance, depending on the extent of deformation, the medium, and the degree of water absorption.^19^ Therefore, the increase in muscle activity may be related to the different vertical dimensions of the aligner after exposure to the buccal environment, and to dental positioning during the orthodontic treatment. In addition, the absence of stable occlusal contacts on the external surfaces of the aligners can be associated with greater muscle activation, possibly attributed to instability and degree of adaptation to using the device, in an attempt to obtain correct jaw positioning during rest. Another aspect worth mentioning is that some participants complained about tooth clenching after orthodontic treatment started. The presence of polyurethane with a hybrid behavior between the teeth prevented the occlusal tooth-to-tooth contact occurring during swallowing, and promoted a clenching habit. Although the lack of tooth-to-tooth contact was not measured in the study, it might have contributed to hypersensitivity of the superficial masseter and the anterior temporal muscles.

Pain is usually referred to as one of the main limiting factors in the bite force of even healthy individuals under test conditions.^20^ ^,^ ^21^ The present results showed a decrease of about 20% in the bite force capacity produced by the superficial masseter and anterior temporal muscles after the orthodontic aligners were installed. A reduction in bite force capacity indicates a possible worsening of the normal masticatory function during MVBF and SVBF. However, the present participants did not report any pain sensation during sEMG acquisition. In this case, it could be hypothesized that an increase in myoelectric activity may have disrupted the 261 muscle synergism affecting force production caused by the aligners in the oral 262 cavity. This likely disruption can be observed when unaccustomed individuals start performing a given task using a more extensive and less coordinated muscle chain, and thus trigger a greater firing rate and number of motor units recruited as a compensatory strategy.^22^ ^,^ ^23^

Hence, the reduction in bite force may refer to specific biomechanical effects that elicit different neural drive strategies on muscle recruitment, evoked by the dental position imposed by each new pair of aligners, even though the subjects did not complain of tooth or muscle pain when acquiring the aligners. Finally, the aligners seem to cause a maladaptive synergism between temporal and masseter muscles when performing biting tasks, thus leading to a reduction in an individual’s maximum ability to exert force between the teeth. 

In this study, the dental and skeletal variables used to select the inclusion and/or exclusion criteria did not contraindicate treatment with orthodontic aligners. However, they were chosen with the goal to establish cases with slight tooth movement since these characteristics may influence masticatory muscle recruitment patterns and function as a confounding factor. It is noteworthy to mention that the absence of a control group with conventional fixed appliances, and of a follow-up during orthodontic treatment were the main limitations of the present study. Nevertheless, in the event of a control group, the biomechanics and the time interval between the application of orthodontic forces would differ between the groups, since the two treatment modalities do not use similar apparatuses. A procedure using both groups would necessarily have various effects on the masticatory muscles, thus invalidating the direct comparison results of this study. 

For this reason, we believe that the present study results were not affected by the lack of a control group, insofar as we did not aim to compare distinct treatment modalities. An ideal control group would be individuals using passive aligners manufactured with SmartTrack^®^ (polyurethane) for the same amount of time as those using activated aligners. However, such a strategy would have ethical implications, because the control participants would be subjected to using a device capable of modifying their salivary composition,^24^ and would thus require complementary dental hygiene practices,^25^ among other aspects. Despite the strategy developed, the present results should be interpreted with caution, and should not be extrapolated to all populations, considering the limited number of participants involved in the study.

## CONCLUSIONS

The present preliminary study with a limited sample revealed that using orthodontic aligners affected changes in recruitment patterns of masticatory muscles and a decrease in bite force capacity during the 8-month follow-up treatment period. The same results were obtained for tasks performed with or without the aligners, indicating that the change in muscle recruitment is prompted by a functional adaptation of the muscles under study, regardless of the occlusal separation caused by the aligners in the mouth. In line with the present objective, we raised awareness of the effects of aligners on masticatory physiology during orthodontic treatment. In conclusion, the risk of increased muscle activity, decreased chewing performance, and possible myofunctional disorders cannot be ruled out.
